# Rational design of metallic nanocavities for resonantly enhanced four-wave mixing

**DOI:** 10.1038/srep10033

**Published:** 2015-05-14

**Authors:** Euclides Almeida, Yehiam Prior

**Affiliations:** 1 Department of Chemical Physics, Weizmann Institute of Science, Rehovot 76100, Israel

## Abstract

Optimizing the shape of nanostructures and nano-antennas for specific optical properties has evolved to be a very fruitful activity. With modern fabrication tools a large variety of possibilities is available for shaping both nanoparticles and nanocavities; in particular nanocavities in thin metal films have emerged as attractive candidates for new metamaterials and strong linear and nonlinear optical systems. Here we rationally design metallic nanocavities to boost their Four-Wave Mixing response by resonating the optical plasmonic resonances with the incoming and generated beams. The linear and nonlinear optical responses as well as the propagation of the electric fields inside the cavities are derived from the solution of Maxwell’s equations by using the 3D finite-differences time domain method. The observed conversion-efficiency of near-infrared to visible light equals or surpasses that of BBO of equivalent thickness. Implications to further optimization for efficient and broadband ultrathin nonlinear optical materials are discussed.

The rational design of optical metamaterials with dimensions smaller than or comparable to the wavelength of light is fundamental for future applications that require miniaturization of photonic devices^1^,^2^,^3^. While the knowledge of the linear optical properties of these materials has advanced tremendously in the last decade, the nonlinear optical properties are known to a lesser extent. The strong intrinsic nonlinear response of noble metals makes them good candidates for nonlinear optical applications at the nanoscale^4^. The electric field enhancement at plasmon resonance frequencies can be used to boost the already strong nonlinear response of the noble metals by many orders of magnitude^5^,^6^. Furthermore, resonant metallic nanostructures can be combined with other nonlinear materials for enhanced nonlinear generation^7^,^8^, opening up a myriad of possibilities for the engineering of very small, efficient and broadband nonlinear materials.

Second harmonic generation (SHG) has been observed in several metallic nanostructures like arrays of nanoparticles^9^ and nanoholes^5^,^10^, gold “nanocups”^11^ and single nanocavities^12^,^13^ of different shapes. As SHG requires lack of spatial symmetry, the signal in these nanostructures is generated only at the interfaces, where the symmetry is broken, decreasing the overall efficiency of SHG. On the other hand, higher odd-order nonlinear phenomena, like third-harmonic generation and four-wave mixing (FWM), can occur in non-centrosymmetric media, and has been proposed for high-sensitivity nonlinear spectroscopy^14^.

There are different strategies to enhance the optical nonlinear generation in nanostructures and metallic surfaces^6^, ^15^,^16^,^17^,^18^. As in any nonlinear process, the signal strongly depends on the intensity of the input beams, and therefore the material must be tailored to obtain a large field enhancement at the fundamental frequencies. Equally important is the optical response of the material at the frequency of the nonlinear signal, which must not absorb or attenuate the signal^10^. In the case of coherent NL processes, like SHG and FWM, phase matching of the involved fields is required, thus the metamaterial must be designed to reduce phase mismatch^18^.

Here we show that efficient FWM generation can be obtained in an array of metallic nanorectangles. Control of the nanocavities geometrical parameters enables the tuning of the optical resonances to the frequencies of the interacting fields to obtain a stronger FWM signal. Extraordinary optical transmission^19^ (EOT) contributes by increasing the local fields inside the nanoholes and facilitating the propagation of the FWM signal. For demonstration purposes, we limited this study to a single geometrical shape (rectangles), kept the fraction of milled area constant and we only tried to optimize a single parameter – the aspect ratio (AR) of the rectangles. Clearly, the parameter space to be optimized can be much larger (shape, area, film thickness, etc.) but as an illustrative example of rational design we preferred to limit the optimization to a single parameter. The design goes through a numerical calculation of the dependence of the FWM signal on the AR using a nonlinear 3D finite differences time-domain (NL-3D-FDTD) model. Following the design, the fabricated optimized shapes are measured experimentally, and the agreement is found to be very good. Finally, an intuitive mode theory which takes into account field distribution and phase matching^10^ is implemented to our case of FWM in nanocavities. The model provides insight into the observed results and suggests strategies for further optimization of efficient nonlinear metamaterials.

## Results

Periodic square arrays of 45 × 45 rectangular holes were milled in a high quality 250 nm thick free-standing gold film using focused ion beam (FIB). The periodicity of the holes within an array was set to 510 nm. For each array, the aspect ratio (AR) of the rectangles is different, but the open area is kept constant and equal to that of square holes of 200 nm × 200 nm (AR = 1). Several configurations were calculated and the corresponding experiments were performed. For each array, the linear transmission spectrum was predicted and measured for different input light polarization. The FWM at *ω*_*FWM*_ = 2*ω*_1_−*ω*_2_ was measured for two different input frequency combinations, one optimized and one not. Linear transmission spectra were calculated by 3D–FDTD calculations carried out with the Lumerical commercial software package^20^ and the nonlinear response was calculated by the same package, based on the NL-3D–FDTD routines (For details of both the experiments and the calculations see the Methods section below).

### Linear transmission

**Figure 1:**
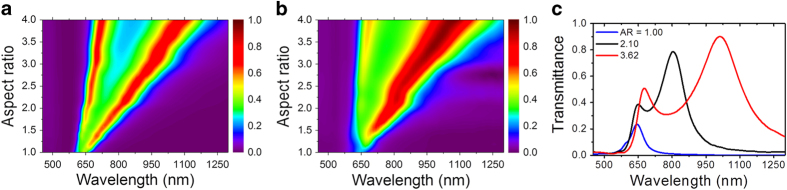
Transmission spectra for different aspect ratios (AR): (**a**) calculated and (**b**) measured spectra covering a wide range of AR; (**c**) Three specific measured spectra for AR = 1.0, 2.1 and 3.6. The calculations were done with coherent white light excitation (see text).

The linear transmission spectra of rectangular cavities, calculated and measured for a range of AR, are shown in Fig. 1a,b. As the aspect ratio increases from AR = 1 to AR = 4, two separate peaks evolve, representing the two accessible modes. The dominant mode, which strongly depends on the AR with transmission peak that changes continuously from 650 to 1100 nm, and a weaker one which hardly depends on the aspect ratio and remains around 650 nm for the entire range of aspect ratios. These two resonances originate with different physical mechanisms. The resonance at longer wavelengths is attributed to localized surface plasmons (LSP) excitation on the nanoholes and therefore strongly depends on their AR^21^,^22^, and like for metallic nanorods, the LSP resonance is red-shifted for more elongated rectangles. The observed peak around 650 nm is commonly referred to as the Fabry-Perot resonance^23^,^24^. Figure. 1c depicts three cross-sections at AR = 1.0; 2.1; 3.6 clearly showing the optimality of AR = 2.1 for resonance excitation at 800 nm. Note that for AR = 1.0 the two modes seem to coalesce, and only a single peak with a slight shoulder is visible.

Several comments are in order:

1. Nanocavities differ from metallic rods in that due to Babinet’s principle, equivalent behavior is seen for polarizations that are rotated by 90 degrees^25^. Thus, in both the calculation and measurements, the light was polarized along the short axis of the rectangle.
2. The transmission spectra were measured with white light which is spatially and temporally incoherent, whereas, in anticipation of the FWM experiment, the calculations presented here were performed with spectrally and spatially coherent light.
3. All the calculations presented in Fig. 1 were carried out for a single “perfect” cavity with perfect walls and theoretically sharp corners. Furthermore, the method of calculation is based on a single cavity with periodic boundary conditions, which thus dictates perfectly reproducible fabrication without any variability between the holes.

A more realistic calculation should allow for cavity dimension variability and less than perfect rectangle corners, and for incoherent light excitation. The numerical approach for performing such a calculation and some typical results are discussed in Supplementary Fig. 1.

**Figure 2:**
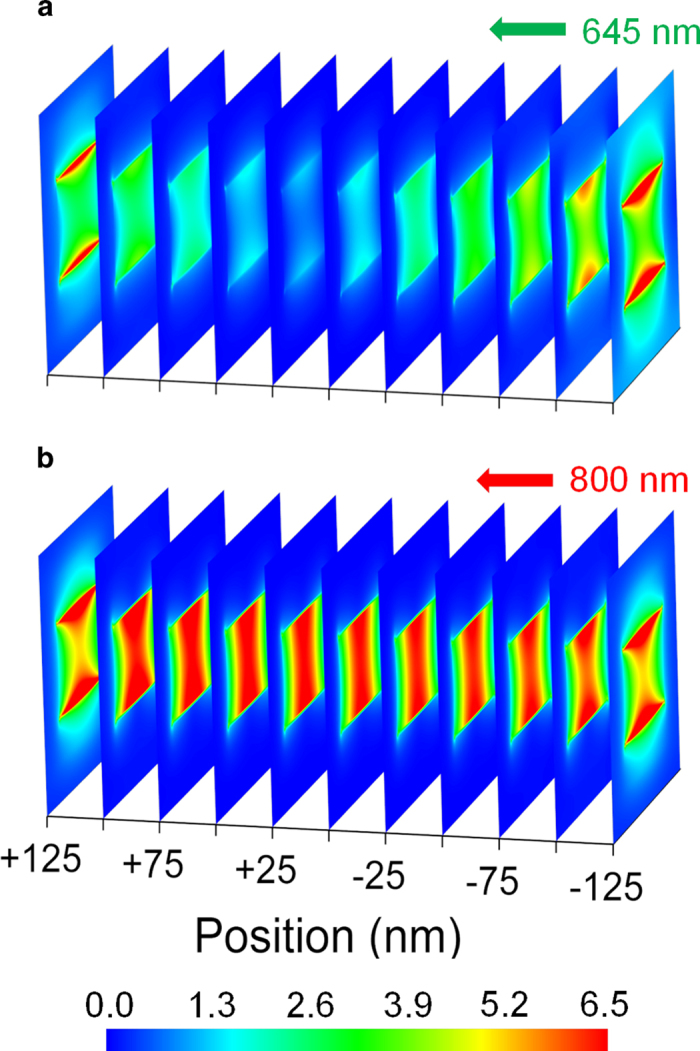
Field distribution for the 645 nm and 800 nm modes propagating down a rectangular nanocavity with dimensions 290 × 138 nm^2^ (AR = 2.1) in a gold film of thickness of 250 nm.

To better visualize the two separate resonances, the calculated distributions of the electric fields at 650 nm and at 800 nm inside the nanocavity are depicted in Fig. 2 for a cavity of 290 × 138 nm^2^ (AR = 2.1). In both cases, light propagates from right to left, and the field distribution profiles (at 645 and at 800 nm) are displayed for increasing propagation distance into the cavity. The difference between the two modes is clear. The FP mode around 645 nm (Fig. 2a) contains a node in the center of the cavity and field maxima at the ends, which is typical to an open-ended Fabry-Perot resonator^23^,^24^. The LSP mode at 800 nm propagates along the cavity with small variations in its transverse profile and shows strong frequency dependence (see Fig. 2b) on the AR. The two propagating modes are visualized in 3D movies in Supplementary Movie 1,2

### Nonlinear Four Wave Mixing

**Figure 3:**
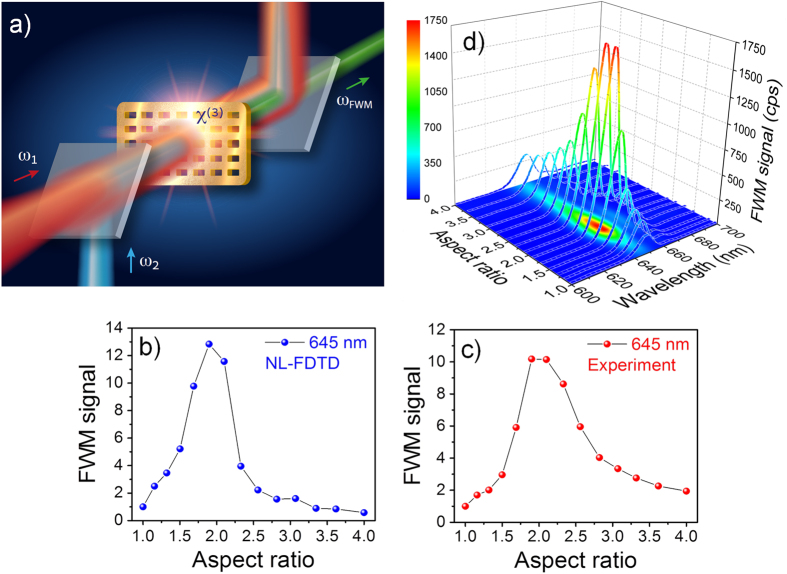
(**a**) Illustration of the FWM processes in an array of rectangular nanoholes. (**b**) Calculated and (**c**) measured AR dependence of the FWM signal at 645 nm and the corresponding (**d**) experimental spectra of the signal.

Using the results of the linear transmittance as the starting point, we formulate a strategy to obtain efficient FWM signal in the visible. The FWM signal (see illustration in Fig. 3) results from the coherent interaction between the two beams via the third-order nonlinear susceptibility *χ*^(3)^:
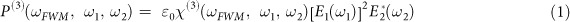
Where *P*^(3)^ is the third-order nonlinear polarization induced in the material and the intensity of the signal is given by 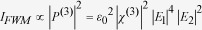. Thus, the product 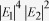 of the input electric fields must be maximized. If we limit ourselves to only a single parameter of optimization, namely the AR, the LSP resonance can be tuned to match one input frequency, and since the dominant contribution comes from the quadratic dependence at *ω*_1_, optimal tuning means matching the LSP resonance to this frequency. We aim for a strong FWM signal in the visible, and this can be achieved at 645 nm, matching the Fabry-Perot mode to obtain enhanced EOT. As shown in the linear calculations and measurements, the LSP resonance should be tuned to match our strong beam at 800 nm (the fundamental frequency of the Ti:Sapphire laser), and this happens for AR = 2.1. The fundamental input frequency *ω*_1_ = 800 nm implies that the second input frequency should be *ω*_2_ = 2*ω*_1_−*ω*_*FWM*_ = 1065 nm.

With the input frequencies determined, we calculate the expected FWM signal for different AR. We use an implementation of a NL-3D-FDTD method using the commercial Lumerical Solutions software^20^. The field update equations of the FDTD algorithm are modified to include a third-order NL polarization induced in a material with instantaneous *χ*^(3)^. Two temporally overlapped 60 fs plane waves centered at frequencies *ω*_1_ and *ω*_2_ were used as the FDTD sources to illuminate a single nanorectangle drilled in a gold film with periodic boundary conditions. The *χ*^(3)^ of gold was chosen according to the value reported in literature^26^ in this spectral region (*χ*^(3)^ = 1 × 10^-18^ m^2^/V^2^). The electric field amplitude of both sources was set to 1 × 10^9^ V/m (equivalent to peak power of 6 × 10^11^  W/cm^2^ or pulse energy of 80 nJ for our pulses and geometry) and the fields are polarized along the short axis of the rectangle. The *z* component of the Poynting vector is spatially integrated on two z-normal planes positioned after (forward FWM) and before (backward FWM) the Au film. The results of this model calculation are depicted in Fig. 3 where the spectrally integrated FWM signal at *ω*_*FWM*_ = 645 nm is plotted against the aspect ratio for input frequencies *ω*_1_ = 800 nm; *ω*_2_ = 1065 nm. A clear resonance is predicted near AR = 2.1 with a very rapid drop for other values, smaller or larger. In particular, a rather weak signal is predicted for a square cavity of AR = 1.

Next, we verified these predictions experimentally. We used two 60 fs pulses, one centered at the fundamental Ti:Sapphire frequency *ω*_1_ = 800 nm, and *ω*_2_ that was obtained from a tunable optical parametric amplifier (OPA) with frequency ranging between 1000 nm to 1300 nm with the same pulse duration and repetition rate (1 KHz). The *ω*_1_ and *ω*_2_ beams are spatially and temporally overlapped at the free-standing gold film in which the nanohole arrays are milled (see Fig. 3). The FWM signal is filtered and measured in both forward and backward directions with a CCD detector coupled to a spectrometer. The use of a free-standing film, in spite of the difficulty in preparing and handling it, is critical for the removal of the much stronger coherent, nonresonant FWM emission, especially for collimated beams where the entire glass thickness contributes. An alternative approach would have been to use tightly focused beams^27^ where only the thickness within the Rayleigh depth contributes. Moreover, in a free standing film, the matching of the refractive index at both interfaces of the arrays^28^ has the added advantage of increasing the observed optical transmittance, higher than >80% in the arrays with large AR. Having similar interfaces on both sides of the film also gives rise to enhanced fields near the surface, but this is outside the scope of the present work.

In the first set of FWM experiments, the wavelength of the OPA was tuned to *ω*_2_ = 1050 nm so that the generated FWM signal at *ω*_*FWM*_ = 645 nm will match the FP cavity resonance which was predicted theoretically and observed experimentally in the linear transmission measurement. Fig. 3 shows the spectrally integrated FWM signal for several different AR along with the corresponding spectra of the FWM signal around 645 nm. As predicted by the NL calculations, shape dependent resonance is seen for rectangles with AR = 2.1, with more than an order of magnitude enhancement for AR = 2.1 as compared to AR = 1.

**Figure 4:**
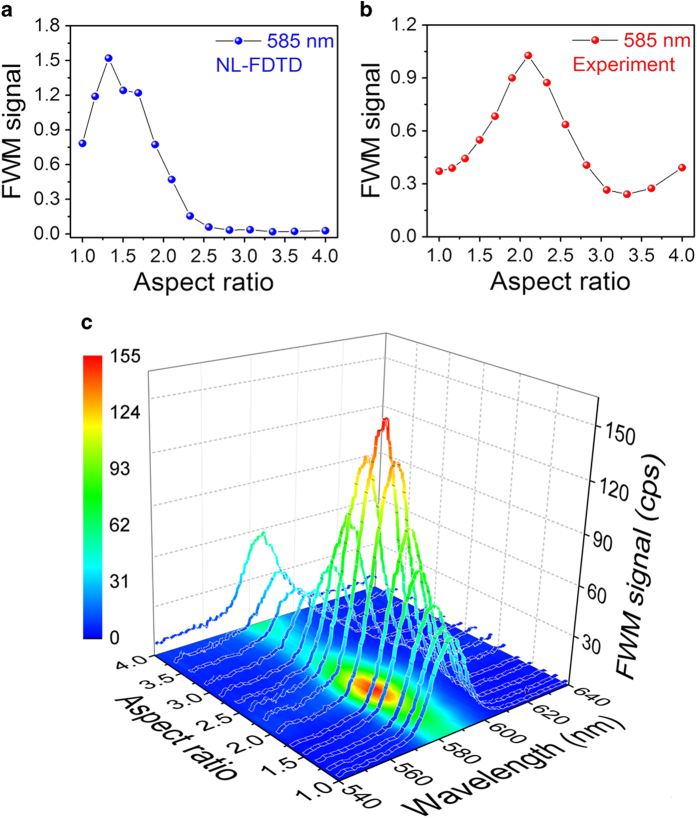
(**a**) Calculated and (**b**) measured AR dependence of the FWM signal at 585 nm. (**c**) Measured spectra of the FWM signal around 585 nm. The FWM intensity was normalized identically to fig. 3, namely the intensity at AR = 1 for the 645 nm case is taken as 1. Thus, the intensities in figs. 3,4 can be directly compared.

To clarify the significance of the FP-resonance for the NL generation, a different input wavelength was chosen (*ω*_2_ = 1265 nm) to generate an unoptimized signal at *ω*_*FWM*_ = 585 nm, which is spectrally removed from the FP resonance. Figure. 4 depicts the calculated and measured AR dependence of the integrated signal. While the FWM signal is still maximal at AR = 2.1, the intensity of the FWM signal at 585 nm is a full order of magnitude weaker. As discussed, the maximum at AR = 2.1 can be traced directly to the LSP resonance at 800 nm, and therefore it is seen in both the optimized FWM at 645 nm, and at the non-optimized FWM at 585 nm. As seen in the linear transmission spectra, and supported by the 3D–FDTD calculations, the field enhancement at *ω*_2_ = 1265 nm is similar to that for *ω*_2_ = 1050 nm (both frequencies are far out of resonance with the LSP mode for AR = 2.1). Therefore, the contribution of the electromagnetic field (EMF) enhancement in eq. (1) is similar for both *ω*_2_ frequencies. Assuming a smooth dispersion relation for *χ*^(3)^, the enhanced FWM at 645 nm may be directly attributed to EOT at the FP resonance.

We have also measured the backwards FWM, and found it to be more than an order of magnitude smaller, because under our experimental conditions, phase matching is more efficient in the forward direction^18^. In all these measurements, the signal from the bare gold film (without holes) is either not visible at all or hardly detectable above the noise level. Note also that the measured shape resonance is broader than the calculated one, its contrast ratio less pronounced and the peak is displaced to slightly lower aspect ratio. These observations are discussed below.

As a last point in this section, we try to estimate the FWM efficiency. When detector efficiency and losses in optical components along the detection path of the signal (estimated to be around 90%) are taken into account, the extracted FWM conversion efficiency is of the order of 10^−8^, which is comparable to the published efficiency of a BBO nonlinear crystal when calculated for the same thickness of 250 nm^29^. This result is promising as the current first attempt to design efficient nonlinear response was based on optimizing only a single parameter, namely the AR. A large number of other parameters can and will be considered, including size and shape of the cavities, fine fabrication details, spacing between them, film properties such a thickness, etc. These optimizations will be the subject of a future publication.

## Discussion

**Figure 5:**
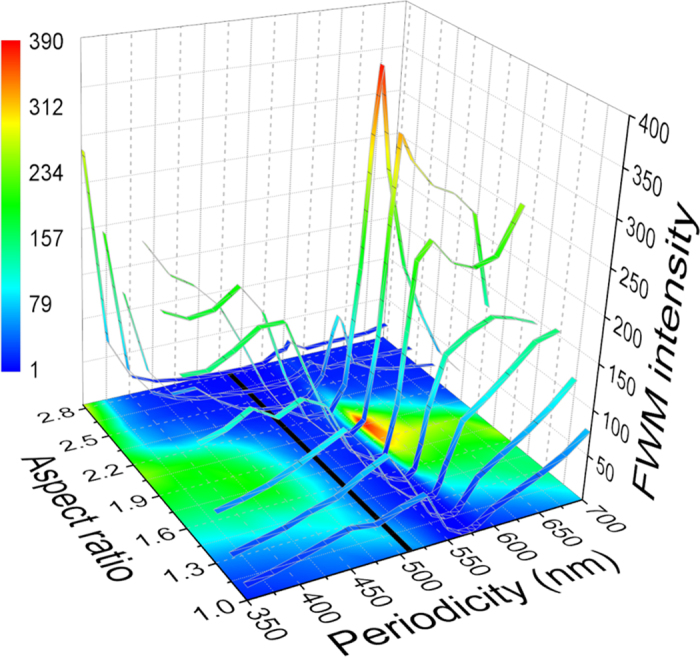
Dependence of the FWM signal at 585 nm on the aspect ratio and the periodicity. For periodicities between 520 and 550 nm, Wood’s anomaly tends to decrease the FWM signal. The black line indicated the periodicity of 510 nm used in our experiments.

The current work is a demonstration that rational design of metallic nanocavities can lead to enhanced, efficient Four Wave Mixing. When designing a strategy for optimizing the shape of nanocavities in a thin film, one may consider many different parameters as variables. These include, among others, film thickness and composition, cavity size and shape (rectangles, triangles, circles or other simple geometrical shapes), shapes with more complex symmetries, coupled cavities of different degree of coupling, and quite a few other parameters. Like many other examples, optimization in a multi-dimensional space is rather complex and one often resorts to computer algorithms and loses physical insight. For this reason we decided to limit ourselves to a single, physical parameter so that the optimization process can be followed and analyzed. Thus, we have focused on rectangles, and optimized their aspect ratio. However, for completeness, we illustrate the very strong dependence of the FWM efficiency on parameters other than the aspect ratio, which in turn can be used for optimization at other wavelengths or under different experimental conditions. Fig. 5 depicts a calculated optimization of the FWM amplitude at 585 nm for arrays of rectangles with varying periodicity and aspect ratio. Clearly, very pronounced dependencies are observed, with the optimal spacing being around 590 nm, and the optimal AR being 2.1 respectively. The FWM amplitude attained with these parameters is approximately 6–fold larger than what was achieved with the previous set of parameters used for the optimization at 645 nm. Note, however, that real optimization must be done in a multidimensional space, optimizing all the parameters simultaneously, and not one parameter at a time, which may only lead to local maxima.

In a related context, we have previously shown that triangular nanocavities of optimized size offer strong enhancement of SHG^13^, and have investigated the coupling of such triangles over large distances^30^. To the best of our knowledge, similar studies have not been reported for higher order nonlinearities^5^,^10^.

**Figure 6:**
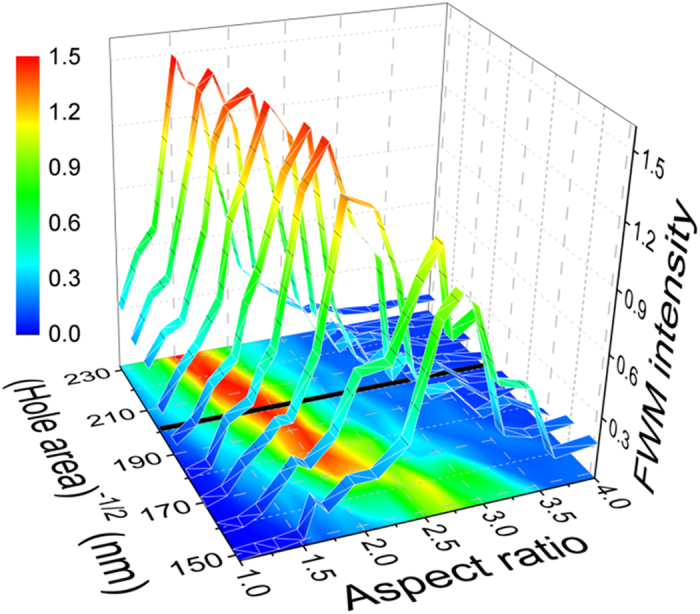
Calculated AR dependence of the integrated FWM signal at 645 nm for different areas defined by a square hole with the shown size. The black line is for 200 × 200 nm^2^ used in our experiments.

In the NL calculations, the FWM resonance appears shifted to slightly smaller AR, and the peak-to-baseline ratio is larger than the experimental value. Moreover, the agreement of the calculated and measured AR dependence of FWM signal at 585 is not very good. We assign this deviation to the existence of the Wood anomaly around 580 nm, a trace of which is visible in the linear transmission spectra in Fig. 1, and which causes a much higher sensitivity to the fabrication parameters. These differences may be caused by inaccuracies in the determination of fabrication parameters such as film thickness and nanocavities’ exact dimensions and shape, FIB resolution, and in particular sharpness (or roundedness) of edges and corners that tends to decrease the field enhancement. To illustrate the influence of these effects and sensitivity of the resonance to exact parameters, in Fig. 6 we show the calculated FWM signal AR dependence for different size cavities. Not only does the peak-to-baseline ratio change, but also the FWM resonance is shifted, depending on the cavity size. Similar observations were previously reported for SHG^10^.

The FWM shape resonance may be investigated analytically using a nonlinear coupled mode theory for energy exchange among the longitudinal modes inside the cavities (Supplementary discussion 1). The analysis is an extension to FWM of the formalism presented in Ref.^10^ for SHG, and as in reference^10^, the generated FWM intensity depends on many factors such as: mode-overlap, phase matching, field enhancement at the input frequencies and attenuation upon propagation of all waves involved. In spite of some inherent limitations, the model provides a semi quantitative analysis and captures the underlying physical mechanism of FWM generation inside nanocavities. As the incoming waves propagate through the cavity, it is found, by the numerical solutions of the propagating waves, that the fields are much stronger inside them than on the surface so that most of the nonlinear interaction takes place, and most of the FWM signal is generated inside the nanoholes. Two longitudinal modes propagate inside the nanoholes, which behave like waveguides. As they propagate, the modes interact with the metal and exchange energy with the FWM mode which builds up. The total generated forward FWM signal is assumed to be sum of the intensity of the FWM modes at the cavity exit.

As anticipated, the predominant factor for the FWM shape resonance is the field enhancement at *ω*_1_ = 800 nm, which exhibits a resonance for AR = 2.1. Although there is a shape resonance in the field enhancement at *ω*_2_ = 1050 nm also for AR = 3.6, it contributes to the FWM signal with the square of the field amplitude, as compared to the stronger, fourth power amplitude dependence of the EF enhancement at *ω*_1_ = 800 nm.

In summary, we demonstrated efficient FWM generation in arrays of periodic nanoholes drilled in a gold film. The FWM efficiency, which is comparable to the conversion efficiency of good nonlinear optical crystals, can be optimized by rational design of the geometry. To demonstrate the utility of the approach, we opted to control only a single parameter, the aspect ratio of the rectangles, and showed that when the cavity resonances match the frequencies involved in the FWM process, significant enhancement is observed. NL–3D–FDTD calculations were implemented that could predict, with good agreement, the shape FWM resonance observed latter in the experiments. A nonlinear coupled mode model was used to explain the FWM generation and the shape resonance in terms of the various individual physical processes that contribute to the FWM generation. We have discussed other parameters that can be optimized, and have included one such example. The control of the processes that lead to strong NL generation in very thin metamaterials is fundamental for the development of the next generation of optical devices, and more work is required in order to explore the very large parameter space available for optimization of specific processes.

## Methods

### Sample Preparation

**Figure 7:**
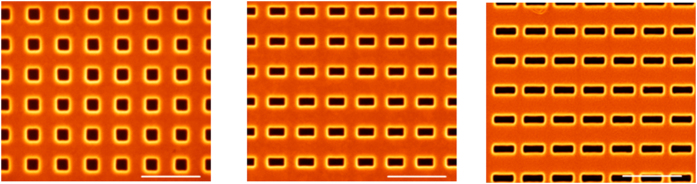
SEM pictures of arrays of rectangles milled in a free-standing, 250 nm thick gold film. Three different aspect ratios are shown (from left to right) AR = 1, AR = 1.5 and AR = 3.1, the scale bar is 1 μm.

Large area, high quality free-standing gold films were prepared by depositing gold on silicon, and etching the silicon from under the gold (Supplementary Fig. 1). The films were characterized by SEM and found to be of very high surface quality. The rectangular nanocavities were milled by a focused ion beam machine (*FEI, Helios Nano Lab 600i*). Various aspect ratio rectangles were milled (Fig. 7), with a constant spacing of 510 nm between them, and for all the arrays, the milled area was kept constant and equal to that of a 200 × 200 nm^2^ square.

### Linear Transmission spectral measurements

The linear transmittance measurements were made using a 20 W Quartz-Tungsten-Halogen Lamp (Newport, 66310 QTH) as the light source. The measurements were carried out for normally incident light. Due to the limited spectral responsivity of the photodector, the spectra were taken in two different spectral ranges. From 450 to 900 nm, the light transmitted through the arrays was detected by a CCD (Jobin-Yvon, Symphony) coupled to a *f* = 190 mm spectrometer (Jobin-Yvon, Triax 180). From 900 to 1350 nm, the transmitted light was modulated and lock-in detected by an InGaAs photodiode (Thorlabs, SM05PD5A) coupled to a spectrometer (SPEX) controlled by a computer. The spectra acquired with the different detection systems were each normalized to the light transmitted through an open area equal to the integrated area of the exposed array, and stitched for complete spectral visualization.

### Linear 3D-FDTD calculations

The 3D-FDTD method (Lumerical Solutions^20^ software package) was used to calculate the linear transmission spectra of the arrays. The dimensions of the fine mesh around the rectangles were set to dx = 2 nm, dy = 2 nm and dz = 5 nm. Here, x, y are the long and short axes of the rectangles, and z is the propagation direction. Periodic boundary conditions were used along the x and y axis while perfectly matched layers were used in the z axis. The light source (plane wave) was polarized along the y axis and traveled with wavevector parallel to the z axis. The dielectric constants of the gold metal were taken from the Palik table^31^. Anticipating the coherent FWM experiments, in the calculation we assume a coherent source also for the linear transmission calculation. However, since most transmission spectral measurements (including ours) are performed with an incoherent white light source, there are differences between the observed and calculated spectra, and these are discussed in Supplementary Fig. 1

### Four Wave Mixing

In the FWM experiment two pulses are used as inputs: the fundamental at *ω*_1_ = 800 nm, generated by a Chirp Pulse Amplified Ti:Sapphire laser (Spectra Physics Spitfire pumped by a Mai Tai), and a pulse from an optical parametric amplifier (OPA) pumped by the same laser and tunable between 1000 nm to 1300 nm. Both pulses operate at a repetition rate of 1 KHz, they are 60 fsec long, of power 68 and 12 μJ respectively. The input pulses are spatially and temporally overlapped at the nanohole arrays. The FWM signal is generated at *ω*_*FWM*_ = 2*ω*_1_−*ω*_2_, the signal is filtered and measured in both forward and backward directions with a CCD detector coupled to a spectrometer (See supplementary fig. 3).

### Nonlinear 3D-NL-FDTD calculations

The 3D nonlinear finite differences time domain (3D-NL-FDTD) simulations were carried out with the same Lumerical Solutions software. The FDTD method solves Maxwell’s equations in discrete time and space (Yee’s cell). As an illustration of the method, consider the simplest 1D case of a TM field propagating along the z direction in a nonmagnetic, nondispersive and homogenous medium. The Maxwell equations are written as:
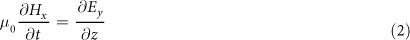

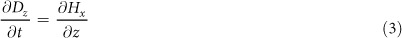
where *E* and *H* are the electric and magnetic fields respectively, *μ*_0_ is the magnetic permeability and 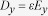 is the displacement field with the frequency-independent electric permittivity 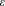. The previous equations can be discretized using the Yee’s algorithm:
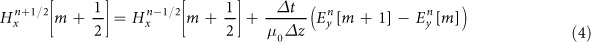

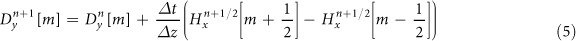

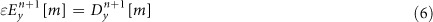
where *n* is the time step and *m* denotes the spatial position of a single cell. The third-order nonlinearity of the material is added in the dielectric constant 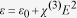, where *χ*^(3)^ is the (nondispersive) third-order susceptibility. Therefore, by modifying the dielectric constant of the material to include the nonlinearity, we can obtain a new update equation for the electric field:
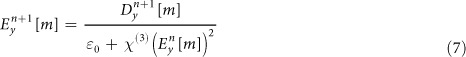


The electric field calculated in the previous time step is used to update the new electric field, which in turn is used to update the new magnetic field (eq. 5) therefore closing the loop. To write eq. 7, the nonlinearity is assumed instantaneous.

In the 3D–NL-FDTD simulations, the dimensions of the mesh around the rectangles were set to dx = 6 nm, dy = 4 nm and dz = 5 nm. We simulate a single rectangle milled in a 250 nm thick gold film and chose periodic boundary conditions with period 510 nm in both x and y directions. Perfectly matched layers were added in the z direction. The dielectric constants were taken from Palik. The diagonal terms of the nonlinear susceptibility were set to *χ*^(3)^ = 1 × 10^-18^ m^2^/V^2^ and the nondiagonal to zero. A y-polarized plane wave centered at *ω*_1_ = 800 nm with pulse duration 60 fs, offset 120 fs and amplitude 1 × 10^9^ V/m is mixed with another plane wave centered at *ω*_2_ = 1050 nm of same pulse duration, offset and amplitude. The two plane waves travel along the z direction and are initially set 150 nm far from the surface. The forward (backward) signal centered at *ω*_*FWM*_ = 645 nm is acquired by spatially integrating the z-component of the Poynting vector in a power monitor located after (before) the metal surface.

## Additional Information

**How to cite this article**: Almeida, E. and Prior, Y. Rational design of metallic nanocavities for resonantly enhanced four-wave mixing. *Sci. Rep.* **5**, 10033; doi: 10.1038/srep10033 (2015).

## Supplementary Material

Supplementary Information

Supplementary Information

Supplementary Information
