# Supplementary material for: Rational design of metallic nanocavities for resonantly enhanced four-wave mixing
Source: Sci Rep. 2015 May 14;5:10033. doi: 10.1038/srep10033 (PMC4650325; doi:10.1038/srep10033)
Supplement: Supplementary Information [file srep10033-s1.pdf]

Supplementary information for

Rational design of metallic nanocavities for resonantly enhanced four-wave mixing

Euclides Almeida and Yehiam Prior

Department of Chemical Physics, Weizmann Institute of Science, Rehovot 76100, Israel

Supplementary Figure 1: Non-uniform sample with spatially incoherent excitation

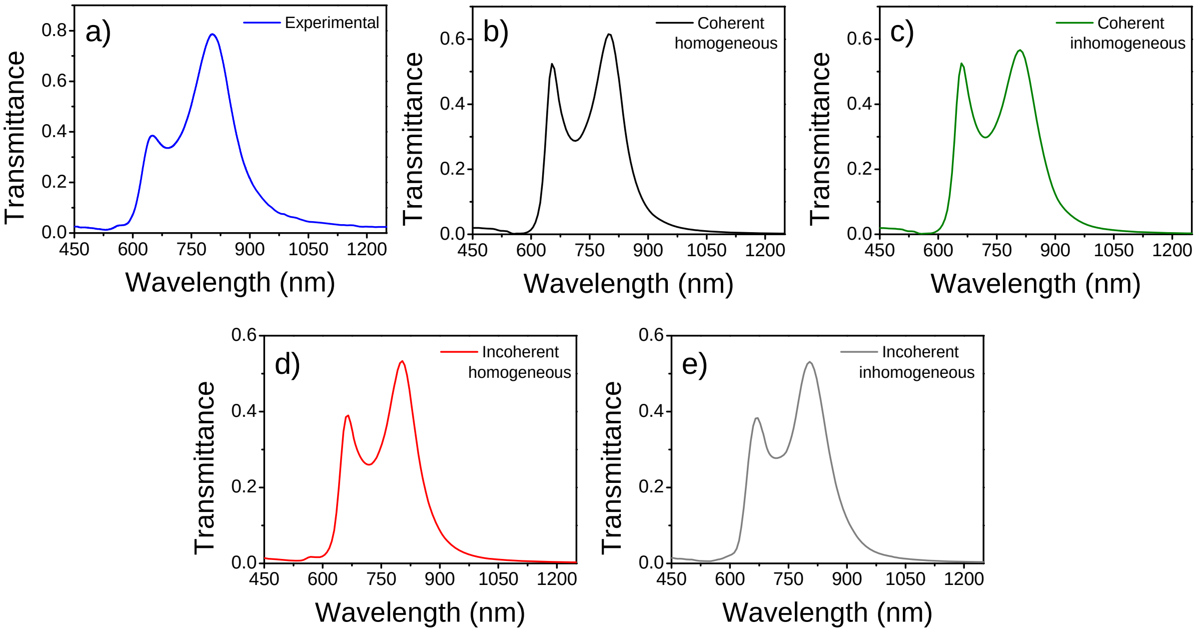

In anticipation of the coherent FWM experiments, and to simulate the laser sources used for these experiments, the linear transmission was calculated using spatially and temporally coherent light source. The linear measurements, however, are based on white light illumination with a source that is neither spatially nor temporally coherent. Furthermore, the calculations are performed on a single cavity, with periodic boundary conditions, which implies a perfect sample where all cavities are identical, obviously unachievable in experimental reality. To test the applicability of these approximations, we repeated some of the calculations for spatially incoherent light, and for nonuniform samples. The light source is simulated as follows: The light

source is divided into 12x12 plane wave sources each one spans 2.1 μm in both x and y directions, each centered on a different hole and having a random phase. Sample nonuniformity is simulated by performing the calculation on an array with 12x12 holes. The periodicity of the square array is maintained at 510 nm, but the dimensions of each hole are allowed to randomly vary (with Gaussian distribution) within 7 nm in x and y around the nominal size of 290x138 nm^2 (AR = 2.1).

a) The experimental transmittance of an array of rectangles

b) Simulated transmittance for coherent excitation and homogeneous sample

c) Simulated transmittance for coherent excitation and inhomogeneous sample

d) Simulated transmittance for incoherent excitation and homogeneous sample

e) Simulated transmittance for incoherent excitation and inhomogeneous sample

Note that the Wood's anomaly, which is a result of long-range coherent interaction (interference), disappears for the simulated spectra of coherent excitation. Note also that for the inhomogeneous samples, linewidths of Fabry-Perot and plasmonic resonances are inhomogenously broadened.

Supplementary Figure 2: Free standing gold films

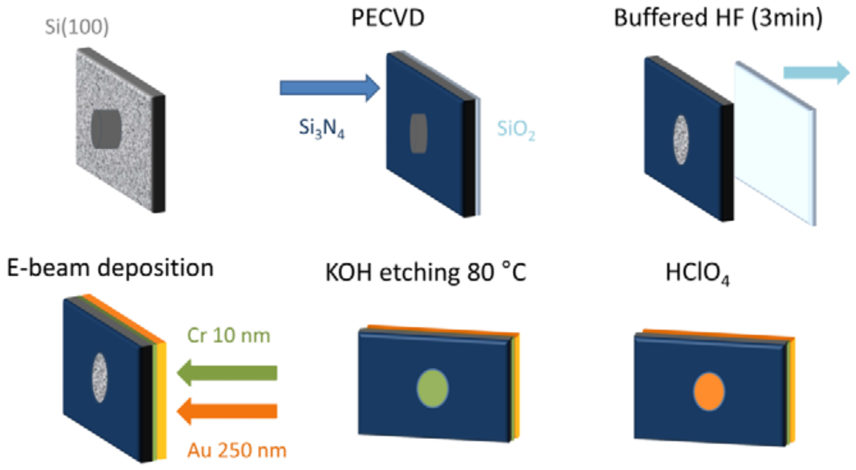

The preparation of free standing gold films was done out as follows: A 5 µm thick Si_3N_4 mask was deposited onto the rough side of a [100] silicon wafer by plasma-enhanced chemical vapor deposition (PECVD). The substrate was immersed in a buffered oxide etch solution for 3 minutes to remove the silica layer formed during the PEVCD process. A 10 nm thick chromium adhesion layer was deposited onto the smooth surface of the wafer by e-beam evaporation, followed by evaporation of a 250 nm thick gold layer. The substrate was then chemically etched by a 45% wt. KOH solution at 80 ºC. The substrate was left in the solution till no more bubbles from the reaction were visible around the area of the hole. A weak stream of distilled water was used to wash the sample after the etching. The chromium layer in the free-standing area was chemically removed by dipping it for a few seconds into $HClO_4$ 37%.

Supplementary figure 3: FWM experimental setup

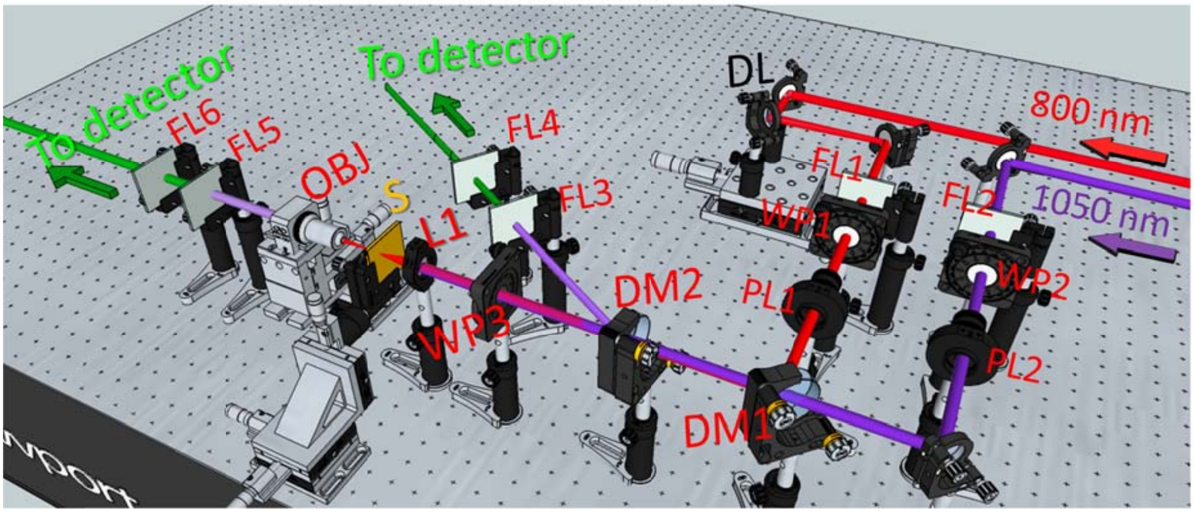

The pulses from an amplified Ti:Sapphire laser ($\omega_1 = 800$ nm, pulse duration = 60 fs, repetition rate = 1 kHz) are used to pump a tunable optical parametric amplifier (OPA) delivering pulses in the NIR with the same repetition rate and pulse duration. In our experiments, the OPA frequency was set to $\omega_2 = 1050$ nm to generate a FWM signal at $\omega_{FWM} = 645$ nm, or to $\omega_2 = 1265$ nm to obtain FWM generation at $\omega_{FWM} = 585$ nm. The optical path of the $\omega_1 = 800$ nm beam can be adjusted by a delay line (DL) to temporally overlap the two beams in the sample. Residual visible light in the ω_1 and the OPA beams is filtered out by longpass filters (FL1 - Thorlabs FEL0750) and (FL2 - Thorlabs FEL0900). The intensity of the two beams arriving in the sample could be controlled by two identical sets of broadband half-wave plates (WP1,2 - Casix, WPZ1315) and polarizer (PL1,2 - Casix, PGL5010). The two beams are combined in the same optical path by a dichroic mirror (DM1 - Thorlabs, DMLP900). The polarization of the two beams could be jointly rotated by a half-wave plate (WP3-Casix, WPZ1315). The beams are focused on the sample (S) by a spherical lens (L1 - focal length 20 mm). The energy per pulse of the beams was set to 68 nJ at 800 nm and 12 nJ at 1050 nm. The diameter of the beams on the sample is 15 µm. The backward FWM signal is reflected at low incidence angle (with respect to the normal) by a laserline filter (DM2 - Laser components, 532NB3) positioned between the dichroic mirror and the half-wave plate, and filtered by a set of shortpass filters (FL3,4 - Thorlabs FESH0750 + Melles Griot 03SWP412). The backward FWM signal is coupled to a

multimode optical fiber and sent to a CCD camera (Jobin Yvon, Symphony) connected to a spectrometer (Jobin Yvon, Triax 180). The forward FWM signal is collected by a 50X objective lens (OBJ - Mitutoyo, 378-811-3) and filtered by shortpass filters (FL5,6 - Thorlabs FESH0750 + Melles Griot 03SWP412). The signal is then coupled to a multimode optical fiber and sent to the spectrometer+CCD system.

Supplementary discussion 1: Nonlinear coupled mode theory simulations

The energy exchange between the longitudinal modes inside the cavities is discussed within the nonlinear coupled mode theory. The model provides a semi quantitative analysis and captures the essence of the underlying mechanism leading to the enhancement of the FWM response. The propagating waves are assumed to be stronger inside the cavity than on its surface so that most of the FWM signal is generated inside the nanoholes, as is seen by the numerical solutions of the wave equations. As the two longitudinal modes propagate inside the nanoholes, they interact with the metal and exchange energy with the generated FWM mode. The observed/measured forward FWM signal is taken as the integrated intensity of the FWM modes at the cavity exit.

The electric field of the FWM mode inside a cavity obeys the wave equation

$$\nabla^2 \boldsymbol{E}_{FWM} - \frac{\epsilon(\omega_{FWM})}{c^2}\frac{\partial^2}{\partial t^2}\boldsymbol{E}_{FWM} = \frac{1}{\varepsilon_0 c^2}\frac{\partial^2}{\partial t^2}\boldsymbol{P}^{(3)} \quad (1)$$

where the nonlinear polarization $\boldsymbol{P}^{(3)}$ is given by eq. (1). The fields propagating inside the cavity can be expressed in terms of the dominant transverse lowest-order mode

$$\boldsymbol{E}_j(x,y,z,t) = A_0^{(j)}\boldsymbol{E}_{j,0}(x,y)e^{i(k_{j,0}z-\omega_j t)} \quad (2)$$

with (j=1, 2 , FWM), $A_0^{(1)}$ and $A_0^{(2)}$ are the amplitude of the fields at z = 0 and $A_0^{FWM} = A_0^{FWM}(z)$ is a slowly varying function of z. The propagation constants $k_{j,0} = k_{j,0}^{real} + ik_{j,0}^{img}$ and $A_0^{(j)}$ can be obtained from the 3D-FDTD calculations. As the incoming fields are polarized along the y axis and hence the fields inside the cavities are mostly polarized in the same axis (according to the FDTD calculations), the tensor product in eq. 1 can be turned into scalar (the diagonal term $\chi_{yyyy}^{(3)} = \chi_d^{(3)}$ of the susceptibility tensor is much larger than the off-diagonal terms). Therefore, eqs. (2) and (1) can be plugged in eq. (1) in the main text, assuming that the FWM signal is generated mostly by Au atoms at the surfaces of the cavities and the FWM amplitude at entrance of the cavities is zero ($A_0^{FWM}(z=0)=0$), we get for the evolution of amplitude of the FWM mode

$$\left|A_0^{(FWM)}(z)\right| = \frac{\omega_{FWM}^2}{2c^2}\chi_d^{(3)}\left|\frac{\sin(\Delta kz/2)}{k_{FWM}\Delta k/2}\right|\left|A_0^{(1)}\right|^2\left|A_0^{(2)}\right|\Theta \tag{3}$$

where the quantity

$$\Theta = \frac{\iint_S \left|E_{1,0}(x,y)\right|^2 E_{2,0}(x,y)E_{FWM,0}(x,y)dxdy}{\iint_S \left|E_{FWM,0}(x,y)\right|^2 dxdy} \tag{4}$$

is called the spatial overlap factor and it is a degree of transverse mode matching between the longitudinal modes. In eq. (4), the integrations are performed on the cavity walls, where the fields are much stronger than anywhere else inside the metal. The phase matching condition $\Delta k = 2k_1 - k_2 - k_{FWM}$ accounts for the momentum conservation of the FWM process.

The intensity of FWM signal is given by the spatial integration of the intensity of the FWM mode at the cavity exit ($z = l$)

$$I_{FWM} \propto \iint_A |E_{FWM}(x,y,l)|^2 dxdy$$

$$= \frac{\omega_{FWM}^4 l^2}{4c^4}\left[\chi_d^{(3)}\right]^2\left|\frac{\sin(\Delta kl/2)}{k_{FWM}\Delta kl/2}\right|^2\left|A_0^{(1)}\right|^4\left|A_0^{(2)}\right|^2|\Theta|^2 e^{-2k_{FWM}^{img}l}\iint_A \left|E_{FWM,0}(x,y)\right|^2 dxdy \tag{5}$$

With the analytical approximation, the FWM process in the nanocavities is now explicitly divided into the several factors that are individually easy to understand. These factors, calculated for the different ARs using the FDTD method, are shown in the figure below. The differences in the propagation constants of the three longitudinal modes (parts a,b) causes a phase mismatch (part c) as the modes propagate inside the cavities. Combined with the attenuation of the FWM mode, it decreases the FWM efficiency. There is no explicit phase-matching resonance for a particular AR. Equally, the transverse mode profile are similar for the different AR and the spatial overlap factor (part d) weakly grows with the AR.

The analytic calculations assumed, for simplicity, that on the average the fields propagate and decay exponentially inside the nanocavities, but this is not fully supported by the numerical calculations. At the FP resonance, for example, the field decays to a minimum value and then grows again. It seems, however, that due to the phase matching, the use of an average value for the field still represents the dependence of the FWM signal on the cavity parameters.

The parameters used in the FDTD simulations used within the couple mode theory calculation are the same used in the linear FDTD simulations except for: (1) The dimensions of the mesh were set to dx = 6 nm, dy = 6 nm and dz = 3 nm for a better accuracy in the propagation direction, and (2) For the light sources we used three narrowband plane waves centered at ω_{FWM} = 645 nm, $\omega_1 = 800$ nm and $\omega_2 = 1050$ nm. The field amplitudes $A_0^{(1)}$ and $A_0^{(2)}$ were calculated in a plane positioned inside the holes 65 nm far from the input side. This avoids numerical artifacts arising due to sharpness of the cavities edges.

To calculate the real part $k_{j,0}^{real}$ of the propagation constant $k_{j,0} = k_{j,0}^{real} + ik_{j,0}^{img}$, two time detectors are positioned 120 nm apart inside the holes. The time spent by the pulses to travel between the detectors is calculated for the first few optical cycles only in order to avoid artifacts due to the multiple reflections inside the cavity. The imaginary part $k_{j,0}^{img}$ is obtained according to $T = (1 - R)e^{-2k_{j,0}^{img}l}$, where T and R are the calculated transmittance and reflectance respectively, and *l* is the cavity length (film thickness).

In conclusion, it is seen that the shape resonance at $\omega_1 = 800$ nm, due to the fourth power dependence on the intensity of this field, is the dominant factor for the FWM field enhancement at AR = 2.

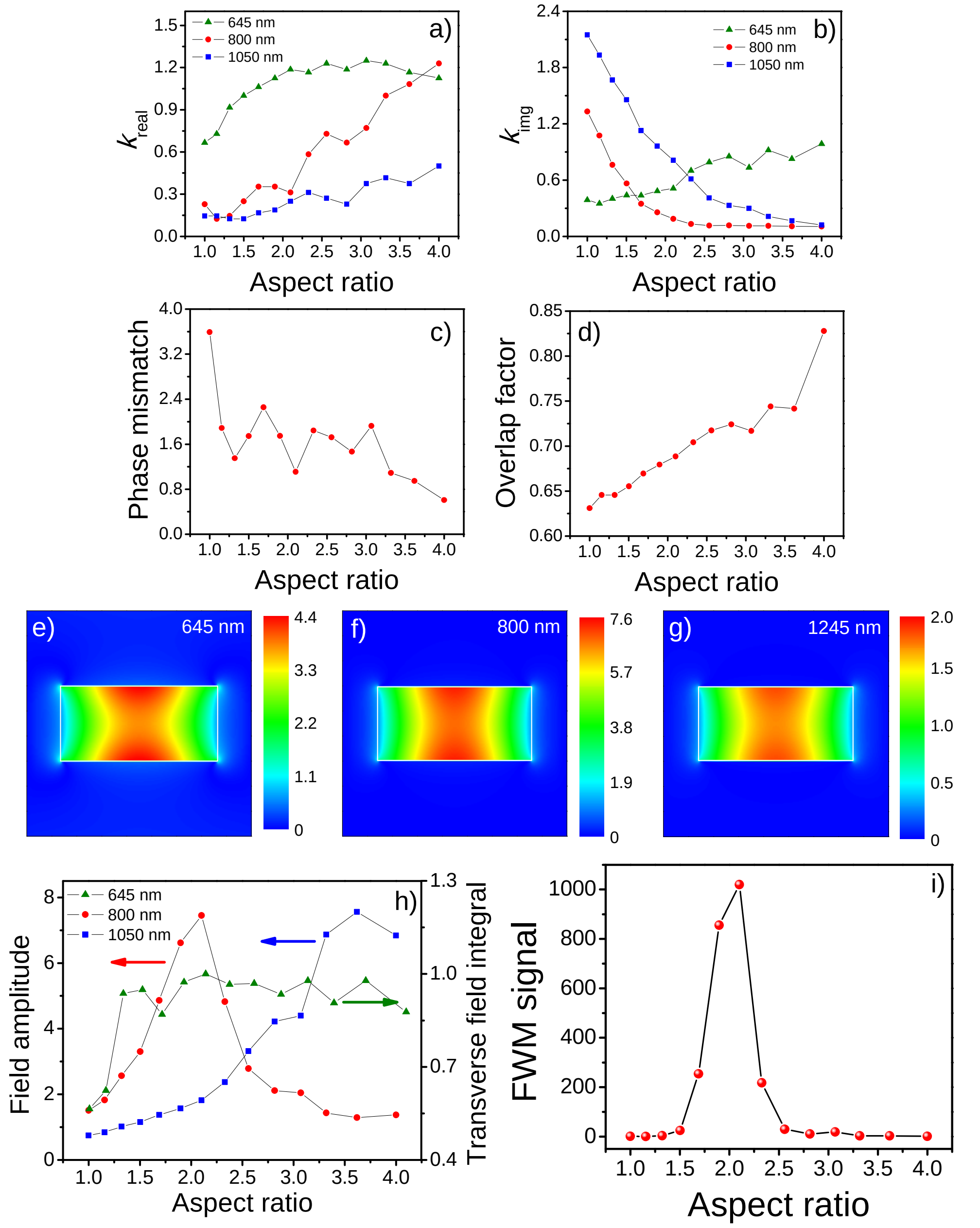

Theoretical results of the FWM shape resonance based on coupled-mode theory. Real (a) and imaginary (b) components of the cavity propagation constants (kl) at ω_{FWM} = 645 nm (olive triangles), ω_1 = 800 nm (red circles) and ω_2 = 1050 nm (blue squares). (c) Phase mismatch factor $|sin(\Delta kl/2)/k_{FWM}(\Delta kl/2)|^2$. (d) Transverse mode overlap factor Θ in eq. 4. (e)-(g) Near-field intensity distribution at ω_{FWM} = 645 nm (e), ω_1 = 800 nm (f) and ω_2 = 1050 nm (g) in the entrance of a rectangle with AR = 2.1 (290x138 nm^2). (h) Field amplitude $A_0^{(j)}$ (j = 1 – red circles, 2 – blue squares) and transverse field integral $\iint_A |E_{FWM}(x,y)|^2 dxdy$ (olive triangles). (i) Calculated FWM signal I_{FWM} according to eq. (5) above.

Supplementary Movie 1,2: comparison of the Fabry Perot and the LSP modes

My video645_wall.avi

My video800_wall.avi
